# Sex‐specific risk factors of carotid atherosclerosis progression in a high‐risk population of cardiovascular disease

**DOI:** 10.1002/clc.23931

**Published:** 2022-10-13

**Authors:** Qi Cheng, Dan Zhou, Jiabin Wang, Zhiqiang Nie, Xiaoxuan Feng, Yuqing Huang, Qiaomin Liang, Yingqing Feng

**Affiliations:** ^1^ Department of Cardiology, Guangdong Cardiovascular Institute, Guangdong Provincial People's Hospital Guangdong Academy of Medical Sciences Guangzhou China; ^2^ Hypertension Research Laboratory, Guangdong Provincial Clinical Research Center for Cardiovascular Disease, Guangdong Cardiovascular Institute, Guangdong Provincial People's Hospital Guangdong Academy of Medical Sciences Guangzhou China; ^3^ Community Health Center of Xiaolan Town Zhongshan China

**Keywords:** atherosclerosis, carotid intima‐media thickness, carotid plaque, risk factors

## Abstract

**Background:**

The progression of carotid intima‐media thickness (cIMT) and plaques are associated with cardiovascular health, especially for high‐risk population of cardiovascular disease (CVD).

**Hypothesis:**

Risk factors for atherosclerosis may vary by sex. This study aimed to investigate the sex‐specific risk factors of cIMT and plaque progression.

**Methods:**

We selected subjects who were identified as high‐risk population of CVD, and collected their carotid ultrasound data and baseline characteristics. Linear regression and logistic regression analyses were used to identify risk factors for cIMT and plaque progression. Sex‐specific risk factors were identified respectively.

**Results:**

A total of 7908 participants were included. The mean age was 57.75 ± 9.45 years and 61.51% were female. During mean follow‐up of 1.92 ± 0.89 years, the median annual cIMT change rate was −7.25 μm/year. Seven hundred and fifteen subjects free from plaques at baseline developed plaque. Age, smoking, hypertension, and diabetes were common risk factors for carotid atherosclerosis progression in all participants. Smoking and alcohol drinking were significantly associated with increased cIMT change in women, while hypertension and antihypertensive medication were significant in men. Increased total cholesterol and diabetes were significantly associated with new plaque presence in women, while smoking, increased triglyceride, and dyslipidemia were significant in men (*p* ˂ .05 for all cases). The association of baseline cIMT and smoking with annual cIMT change rate and increased total cholesterol with new plaque presence were significantly differentiated between both sexes (*p* for interaction ˂ .05).

**Conclusions:**

The risk factors for cIMT and plaque progression differed by sex.

## 1 INTRODUCTION

Atherosclerotic cardiovascular disease (ASCVD), caused by atherosclerosis, is the leading cause of disability and mortality in middle‐aged and elderly population.^1^ Since the carotid arteries are easy to be assessed, carotid ultrasound, a noninvasive examination method, has been widely used to detect new‐formed atherosclerosis. Early atherosclerosis generally starts with the increased thickness of arterial intima and then the formation of atherosclerotic plaques, which may not cause any perceptible symptoms. The artery stenosis caused by large plaques and the rupture or hemorrhage of the vulnerable plaques is the main pathological mechanisms of further ASCVD like acute coronary syndrome and stroke.^2^ Thus, carotid intima‐media thickness (cIMT) and plaques are valuable indicators to predict cardiovascular and cerebrovascular events.^3^, ^4^ It is important to conduct carotid ultrasound in individuals at high risk of cardiovascular disease (CVD) regularly. The global prevalence of increased cIMT and carotid plaque were estimated to be 27.6% and 21.1% among 30–79‐year‐olds, respectively.^5^ It indicates the existence of carotid atherosclerosis burden worldwide. Epidemiological reports have shown that in healthy population, mean common cIMT is about 0.7 mm, and increased by 10 µm/year, while increased by 20 µm/year in patients with untreated ASCVD.^6^ Interventions may slow or reverse this progression, and further reduce the risk of CVD events.^7^, ^8^, ^9^ Moreover, men and women have different morphology and composition of carotid atherosclerosis, indicating the requirement for sex‐specific prevention and management strategies.^10^ Still, the studies that investigate the sex‐specific risk factors of carotid atherosclerosis are limited. This study intended to detect the risk factors of carotid IMT and plaque progression in a population at high‐risk of CVD and identify the differences by sex.

## 2 METHODS

### 2.1 Study design and population

The study data were applied from the Early Screening and Comprehensive Intervention Program for High‐Risk Population of Cardiovascular Disease, which is a pivotal section of the China‐PEACE (Patient Centered Evaluative Assessment of Cardiac Events) Million Persons Project (Trial Registration Number NCT02536456), aiming at detecting subjects at high risk of CVD to provide early prevention and intervention.^11^ The subject would be defined as having high CVD risk if meeting at least one of the criteria as follow: (1) established CVD; (2) elevated blood pressure; (3) dyslipidemia; (4) 10‐year CVD risk ≥ 20%.^12^

We screened 19,059 subjects between 35 and 75 years old with baseline carotid ultrasound examination data from 1 January 2017 to 31 December 2021 in Guangdong province. After excluding 10,935 subjects missing follow‐up carotid ultrasound data, 161 subjects without complete baseline demographic and clinical data, and 37 subjects with invalid carotid ultrasound data, 7908 participants were enrolled in formal analysis (Supporting Information: Figure S1). The enrolled participants had completed the carotid ultrasound examination twice with the interval longer than 1 year. The study protocol has been adopted by the Ethics Committee of Guangdong Provincial People's Hospital and all participants have submitted informed consent.

### 2.2 Assessment of cIMT and carotid plaque

We evaluated carotid atherosclerosis by measuring cIMT and plaques. The carotid ultrasound examinations were all conducted by trained ultra‐sonographers using standardized methods. We applied Vivid‐S6 (GE Medical System) with a 3–12 MHz phased‐array probe and linear array transducer. The examiner scanned longitudinally through the carotid arteries and bulbs of the participants. Meanwhile, the bilateral values of proximal, middle, and distal thickest cIMT would be recorded as measured from the media‐adventitia interface to the intima lumen interface. The mean thickness of bilateral cIMT was calculated as the average of these values. If the protuberant intima‐media was found, the observer will adjust the direction of the probe to observe the shape and size from multiple angles. The thickness of the plaque was measured through the largest section that can be observed. All original images of measured values are saved. The definition of increased cIMT was a thickness > 0.9 mm,^13^, ^14^ while carotid plaque was defined as cIMT ≥ 1.5 mm or any focal thickening that encroaches into the lumen ≥ 0.5 mm or ≥ 50% of the adjacent cIMT.^15^

### 2.3 Covariate data collection

We applied standardized questionnaires to assess socio‐demographic information (such as age, sex), lifestyle behaviors (such as smoking and alcohol drinking history), combined disease history (including hypertension, diabetes, dyslipidemia, coronary heart disease, myocardial infarction, stroke) and medication history (including antihypertensive, hypoglycemic, and lipid‐lowering medication) of the participants. CVD was defined as having coronary heart disease or having a history of myocardial infarction or stroke. Body mass index (BMI) was calculated by body mass (kg) divided by the square of height (m^2^). Overweight and obesity were defined as BMI 24–27.9 kg/m^2^ and ≥28 kg/m^2^, respectively.^16^ Waist circumference was obtained by using tape to surround the abdomen horizontally with the specific positioning point at the mid‐point line of the anterior superior iliac crest to the inferior margin of the twelfth rib. The systolic blood pressure (SBP) and diastolic blood pressure (DBP) should be measured in resting sitting position at least twice, and the average value was taken. Blood samples were collected in fasting state to test the blood glucose and lipid profile. Low‐density lipoprotein cholesterol (LDL‐C) was calculated by Friedewald formula using the values of total cholesterol (TC), high‐density lipoprotein cholesterol (HDL‐C) and triglyceride (TG). Nonhigh‐density lipoprotein cholesterol (non‐HDL‐C) was calculated as TC subtracting HDL‐C. Hypertension was defined as SBP/DBP ≥ 140/90 mmHg, current antihypertensive medication, or self‐reported. According to blood pressure levels, hypertension was classified into grade 1 (SBP ≥ 140 mmHg and/or DBP ≥ 90 mmHg), grade 2 (SBP ≥ 160 mmHg and/or DBP ≥ 100 mmHg), and grade 3 (SBP ≥ 180 mmHg and/or DBP ≥ 110 mmHg).^17^ Diabetes was defined as fasting blood glucose ≥7.0 mmol/L, current hypoglycemic medication, or self‐reported.^18^ Dyslipidemia was defined as using lipid‐lowering drugs, self‐reported, TC, LDL‐C, TG increased or HDL‐C decreased, TC < 5.2 mmol/L and TG < 1.7 mmol/L were appropriate levels.^19^

### 2.4 Statistical analysis

Continuous variables were presented as mean ± standard deviation or median (25th quartile, 75th quartile) as appropriate. Categorical variables were presented as frequency with percentages. The baseline characteristics and carotid ultrasound data of all participants were stratified into two groups by sex. The categorical variables were compared using *χ* ^2^ test or Fisher exact test, continuous variables were compared by *t*‐test or Kruskal–Wallis *H*‐test. The annual cIMT change rate was calculated by subtracting baseline mean bilateral cIMT from follow‐up mean bilateral cIMT and dividing the individual follow‐up time. New carotid plaque presence was defined as having diagnosed carotid plaque at follow‐up but no plaque at baseline. Correlations between baseline characteristics and annual cIMT change rate were examined by linear regression analyses and performed with two models, using the enter method and excluding variables with multicollinearity. Model I was crude model with no adjusted variables. Model II was adjusted for traditional factors for carotid atherosclerosis (age, sex, smoking, TC, TG, hypertension, diabetes, dyslipidemia, CVD, and lipid‐lowering medication) and significant variables that in the crude model (*p*‐value < .05). Univariate and multivariate logistic regression analyses with backward stepwise method were applied to identify independent risk factors for new carotid plaque formation in participants with no plaque at baseline. The results in adjusted model of linear regression and multivariate logistic regression varied by sex were evaluated, considering the interaction terms. Statistical analyses were conducted using SPSS (version 27.0), R Software and EmpowerStats (X&Y Solutions). *p*‐value < .05 indicated statistic significant.

## 3 RESULTS

### 3.1 Baseline and follow‐up descriptive data

A total of 7908 participants were included. The mean age was 57.75 ± 9.45 years and 4864 (61.51%) of individuals were females. Characteristics of all participants and stratified by sex were presented in Table 1. Overall, 4883 (61.75%) of the participants had hypertension, 1712 (21.65%) had diabetes and 3391 (42.88%) had dyslipidemia. Three hundred and twenty‐two 4.07% of the participants had a history of CVD. The differences were significant between men and women, except for age, obesity, glucose, antihypertensive medication and lipid‐lowering medication. Men were more likely to be overweight, current smokers and drinkers, and had higher blood pressure, higher triglyceride but lower cholesterol, more combined diseases. During 1.92 ± 0.89 years of follow‐up, the median annual cIMT change rate of all participants was −7.25 (25th quartile, 75th quartile: −46.67, 37.04) μm/year. 715 (9.04%) subjects without plaque at baseline developed new plaque. The cIMT and plaque progression between sexes were not significantly distinguished. Additionally, compared to participants without plaque, participants with plaque at baseline aged older, more likely to be males and smokers, and had higher blood pressure, higher atherosclerotic cholesterol and triglyceride, more combined diseases and medication usage (Supporting Information: Table S1).

**Table 1:** Characteristics of the participants

Variables		Sex		
	Total	Male	Female	*p*‐value
Number (%)	7908 (100)	3044 (38.49)	4864 (61.51)	
Demographics and Anthropometrics				
Age, years	57.75 ± 9.45	57.59 ± 9.83	57.85 ± 9.20	.231
Smoking, *n* (%)	1405 (17.77)	1355 (44.51)	50 (1.03)	<.001
Alcohol drinking, *n* (%)	470 (5.94)	423 (13.90)	47 (0.97)	<.001
BMI (kg/m^2^)	24.84 ± 3.41	25.09 ± 3.26	24.69 ± 3.50	<.001
Waist circumference (cm)	86.19 ± 9.51	89.15 ± 8.86	84.34 ± 9.44	<.001
Overweight, *n* (%)	4617 (58.38)	1918 (63.01)	2699 (55.49)	<.001
Obesity, *n* (%)	1310 (16.57)	529 (17.38)	781 (16.06)	.124
Hemodynamics				
SBP (mmHg)	142.32 ± 22.75	144.06 ± 21.83	141.23 ± 23.25	<.001
DBP (mmHg)	83.25 ± 13.19	86.33 ± 13.30	81.32 ± 12.74	<.001
Heart rate (bpm)	78.64 ± 11.06	78.09 ± 11.41	78.99 ± 10.82	<.001
Lipids and glucose (mmol/L)				
TC	5.51 ± 1.49	5.01 ± 1.43	5.82 ± 1.44	<.001
LDL‐C	3.28 ± 1.31	2.92 ± 1.27	3.50 ± 1.29	<.001
HDL‐C	1.48 ± 0.48	1.29 ± 0.43	1.60 ± 0.47	<.001
TG	1.88 ± 1.09	2.00 ± 1.21	1.81 ± 1.01	<.001
Non‐HDL‐C	4.03 ± 1.37	3.72 ± 1.34	4.22 ± 1.35	<.001
Glucose	6.13 ± 1.80	6.13 ± 1.88	6.12 ± 1.76	.801
Comorbidities, *n* (%)				
Hypertension	4883 (61.75)	2018 (66.29)	2865 (58.90)	<.001
Classification of hypertension				<.001
Grade 1	1207 (15.26%)	491 (16.13%)	716 (14.72%)	
Grade 2	2758 (34.88%)	1122 (36.86%)	1636 (33.63%)	
Grade 3	377 (4.77%)	196 (6.44%)	181 (3.72%)	
Diabetes	1712 (21.65)	703 (23.09)	1009 (20.74)	.014
Dyslipidemia	3391 (42.88)	958 (31.47)	2433 (50.02)	<.001
Coronary heart disease	190 (2.40)	122 (4.01)	68 (1.40)	<.001
History of myocardial infarction	80 (1.01)	43 (1.41)	37 (0.76)	.005
History of stroke	137 (1.73)	75 (2.46)	62 (1.27)	<.001
CVD	322 (4.07)	193 (6.34)	129 (2.65)	<.001
Treatment, *n* (%)				
Antihypertensive medication	2510 (31.74)	989 (32.49)	1521 (31.27)	.257
Hypoglycemic medication	688 (8.70)	302 (9.92)	386 (7.94)	.002
Lipid‐lowering medication	408 (5.16)	172 (5.65)	236 (4.85)	.118
Baseline cIMT and plaques				
Baseline mean thickness of bilateral cIMT (mm)	0.77 ± 0.15	0.80 ± 0.15	0.75 ± 0.14	<.001
Baseline increased cIMT *n* (%)	2125 (26.87)	1033 (33.94)	1092 (22.45)	<.001
Baseline plaque presence *n* (%)	3823 (48.34)	1709 (56.14)	2114 (43.46)	<.001
Baseline plaque thickness on the left side (mm)	2.16 ± 1.01	2.26 ± 1.16	2.09 ± 0.84	<.001
Baseline plaque thickness on the right side (mm)	2.17 ± 0.93	2.27 ± 1.06	2.09 ± 0.79	<.001
Follow‐up cIMT and plaques				
Follow‐up mean thickness of bilateral cIMT (mm)	0.75 ± 0.15	0.79 ± 0.16	0.73 ± 0.14	<.001
Follow‐up increased cIMT, *n* (%)	1934 (24.46)	980 (32.19)	954 (19.61)	<.001
Follow‐up plaque presence, *n* (%)	3511 (44.40)	1548 (50.85)	1963 (40.36)	<.001
Follow‐up plaque thickness on the left side (mm)	2.13 ± 0.82	2.24 ± 0.90	2.04 ± 0.74	<.001
Follow‐up plaque thickness on the right side (mm)	2.11 ± 0.75	2.20 ± 0.82	2.03 ± 0.68	<.001
cIMT and plaque progression				
Annual cIMT progression rate (μm/year)	−7.25 (−46.67, 37.04)	−5.21 (−46.88, 38.46)	−9.80 (−46.67, 35.71)	.342
Having newly diagnosed plaque, *n* (%)	715 (9.04)	250 (8.21)	465 (9.56)	.089

*Note*: Values are presented as mean ± standardized differences, median (25th quartile, 75th quartile) or *n* (%).

Abbreviations: BMI, body mass index; cIMT, carotid intima‐media thickness; CVD, cardiovascular disease; DBP, diastolic blood pressure; HDL‐C, high‐density lipoprotein cholesterol; LDL‐C, low‐density lipoprotein cholesterol; n, number; NA, not available; non‐HDL‐C, non‐high‐density lipoprotein cholesterol; SBP, systolic blood pressure; TC, total cholesterol; TG, triglyceride.

### 3.2 Risk factors for cIMT progression

As shown in Table 2, after adjustment in Model II, age, males, smoking, alcohol drinking, DBP, hypertension, diabetes and CVD were significant risk factors for annual cIMT progression. CVD, Alcohol drinking and smoking increased the annual cIMT change rate by 12.338 μm/year (95% CI: 2.709, 21.966), 9.276 μm/year (95% CI: 1.083, 17.469) and 6.925 μm/year (95% CI: 1.114, 12.736), respectively. The association of hypertension with cIMT change rate was inverse in Model I, but it became positive in Model II (*β* = 6.708, 95% CI: 0.274,13.142). Diabetes was negatively related with cIMT change rate in the crude model, but became positively related after adjustment (*β* = 4.824, 95% CI: 0.163, 9.484). Baseline mean bilateral cIMT and TG was inversely associated with cIMT change. Per 0.1 mm cIMT increment in baseline was associated with a cIMT change rate of −28.894 μm/year (95% CI: −30.379, −27.409). Compared to men, women had significant lower cIMT change rate (*β* = −15.484, 95% CI: −20.224, −10.743).

**Table 2:** Linear regression analysis for annual cIMT change rate

Variables	Annual cIMT change rate			
	Model I		Model II	
	β (95% CI)	*p*‐value	β (95% CI)	*p*‐value
Age (per 1 year)	**−0.569 (−0.781, −0.358)**	**<.001**	**1.473 (1.238, 1.708)**	**<.001**
Sex‐female	−2.128 (−6.242, 1.986)	.311	**−15.484 (−20.224, −10.743)**	**<.001**
Baseline mean bilateral cIMT (per 0.1 mm)	**−22.183 (−23.441, −20.926)**	**<.001**	**−28.894 (−30.379, −27.409)**	**<.001**
Smoking	0.452 (−4.785, 5.690)	.866	**6.925 (1.114, 12.736)**	**.020**
Alcohol drinking	1.060 (−7.407, 9.528)	.806	**9.276 (1.083, 17.469)**	**.027**
BMI (per 1 kg/m^2^)	**−1.347 (−1.933, −0.762)**	**<.001**	−0.096 (−0.668, 0.475)	.741
SBP (per 1 mmHg)	**−0.143 (−0.231, −0.056)**	**.001**	0.117 (−0.013, 0.246)	.078
DBP (per 1 mmHg)	−0.047 (−0.199, 0.105)	.541	**0.402 (0.202, 0.601)**	**<.001**
TC (per 1 mmol/L)	−0.431 (−1.776, 0.913)	.529	2.001 (−0.042, 4.044)	.055
TG (per 1 mmol/L)	**−2.140 (−3.970, −0.310)**	**.022**	**−2.217 (−4.003, −0.432)**	**.015**
Hypertension	**−8.063 (−12.179, −3.948)**	**<.001**	**6.708 (0.274, 13.142)**	**.041**
Classification of hypertension^a^		**.015**		**.002**
Grade 1	**−9.476 (−15.400, −3.551)**	**.002**	**6.283 (0.623, 11.943)**	**.030**
Grade 2	**−6.674 (−11.185, −2.162)**	**.004**	3.843 (−0.982, 8.669)	.119
Grade 3	−1.288 (−10.923, 8.347)	.793	**19.875 (10.611, 29.140)**	**<.001**
Diabetes	−3.350 (−8.211, 1.510)	.177	**4.824 (0.163, 9.484)**	**.043**
Dyslipidemia	−2.073 (−6.118, 1.972)	.315	1.568 (−4.723, 7.858)	.625
CVD	0.254 (−9.876, 10.384)	.961	**12.338 (2.709, 21.966)**	**.012**
Antihypertensive medication	**−10.226 (−14.521, −5.931)**	**<.001**	−4.696 (−9.573, 0.181)	.059
Lipid‐lowering medication	−7.030 (−16.079, 2.020)	.128	−6.276 (−15.987, 3.435)	.205

*Note*: Model I: adjust for none; Model II: adjust for age, sex, baseline mean bilateral cIMT, smoking, BMI, SBP, lipids (TC and TG), comorbidities (hypertension, diabetes, dyslipidemia, and CVD), and medicine use (antihypertensive medication and lipid‐lowering medication). Bold values represent statistic significant *p*‐value < .05.

Abbreviations: BMI, body mass index; CI, confidence interval; cIMT, carotid intima‐media thickness; CVD, cardiovascular disease; DBP, diastolic blood pressure; SBP, systolic blood pressure; TC, total cholesterol; TG, triglyceride.

^a^ The association of classification of hypertension with annual cIMT change rate was analyzed with all the variables mentioned in Model II adjusted expect for hypertension and SBP, using normotension as the reference.

### 3.3 Risk factors for new plaque formation

A total of 4085 participants who have no plaque presence at baseline were enrolled in the logistic regression analysis (Table 3). According to multivariate logistic regression model, smoking was associated with an odds ratio of 1.728 (95% CI: 1.366, 2.186). TC ≥ 5.2 mmol/L (OR = 1.653, 95% CI: 1.382, 1.976), hypertension (OR = 1.388, 95% CI: 1.125, 1.712), dyslipidemia (OR = 1.268, 95% CI: 1.029, 1.563) and age (OR = 1.068, 95% CI: 1.056, 1.080) were also significant risk factors for new plaque presence.

**Table 3:** Univariate and multivariate logistic regression analysis for new carotid plaque presence

Variables	New carotid plaque presence			
	Univariate logistic regression		Multivariate logistic regression	
	OR (95% CI)	*p*‐value	OR (95% CI)	*p*‐value
Age (per 1 year)	**1.068 (1.056, 1.080)**	**<.001**	**1.068 (1.056, 1.080)**	**<.001**
Sex‐female	0.871 (0.699, 1.084)	.216		
Baseline mean bilateral cIMT (per 0.1 mm)	0.938 (0.872, 1.010)	.089	0.940 (0.874, 1.011)	.094
Smoking	**1.612 (1.218, 2.134)**	**<.001**	**1.728 (1.366, 2.186)**	**<.001**
Alcohol drinking	0.888 (0.586, 1.344)	.573		
Overweight, *n* (%)	0.932 (0.781, 1.113)	.438		
TC ≥ 5.2 mmol/L	**1.563 (1.237, 1.974)**	**<.001**	**1.653 (1.382, 1.976)**	**<.001**
TG ≥ 1.7 mmol/L	1.186 (0.997, 1.410)	.054	1.175 (0.990, 1.395)	.065
Hypertension	**1.437 (1.158, 1.782)**	**.001**	**1.388 (1.125, 1.712)**	**.002**
Classification of hypertension^a^		**.018**		.078
Grade 1	1.255 (0.973, 1.619)	.081	1.212 (0.942, 1.560)	.135
Grade 2	**1.364 (1.093, 1.703)**	**.006**	**1.260 (1.022, 1.553)**	**.030**
Grade 3	**1.641 (1.077, 2.499)**	**.021**	1.484 (0.987, 2.231	.058
Diabetes	**1.262 (1.023, 1.558)**	**.030**	**1.268 (1.029, 1.563)**	**.026**
Dyslipidemia	1.143 (0.902, 1.449)	.269		
CVD	1.165 (0.702, 1.933)	.555		
Antihypertensive medication	1.201 (0.962, 1.501)	.106	1.233 (0.992, 1.533)	.059
Lipid‐lowering medication	1.189 (0.773, 1.828)	.431		

*Note*: The logistic regression model was adjusted for age, sex, baseline mean bilateral cIMT, smoking, alcohol drinking, overweight, TC ≥ 5.2 mmol/L, TG ≥ 1.7 mmol/L, comorbidities (hypertension, diabetes, dyslipidemia, and CVD) and medicine use (antihypertensive medication and lipid‐lowering medication). Bold values represent statistic significant *p*‐value < .05.

Abbreviations: CI, confidence interval; cIMT, carotid intima‐media thickness; CVD, cardiovascular disease; OR, odd ratio; TC, total cholesterol; TG, triglyceride.

^a^ The association of hypertension classification with new carotid plaque presence was analyzed separately with all the variables mentioned above adjusted expect for hypertension, using normotension as the reference.

#### 3.3.1 Association between risk factors and carotid atherosclerosis progression by sex

According to Table 4, age and DBP were significant risk factors for both sexes. Baseline cIMT had an inverse association with cIMT progression, and it was significantly stronger in women than men (*p* for interaction =  0.001). Smoking (*β* = 38.717, 95% CI: 16.338, 61.096), alcohol drinking (*β* = 27.779, 95% CI: 4.458, 51.100) and SBP (*β* = .168, 95% CI: 0.008, 0.328) were significant risk factors in women, while hypertension (*β* = 12.791, 95% CI: 2.127, 23.456) and antihypertensive medication (*β* = −8.012, 95% CI: −15.642, −0.381) were significant in men. Compared with men, smoking significantly accelerated cIMT progression in women (*p* for interaction = 0.004). Other included variables were not significantly associated with cIMT change in both sexes.

**Table 4:** Association between risk factors and cIMT progression by sex

Variables	Annual cIMT change rate				
	Male		Female		
	β (95% CI)	*p*‐value	β (95% CI)	*p*‐value	*p* for interaction
Age (per 1 year)	**1.513 (1.156, 1.870)**	**<.001**	**1.442 (1.140, 1.744)**	**<.001**	.762
Baseline mean bilateral cIMT (per 0.1 mm)	**−26.465 (−28.554, −24.376)**	**<.001**	**−30.686 (−32.524, −28.847)**	**<.001**	**.001**
Smoking	4.319 (−1.677, 10.316)	.158	**39.418 (16.164, 62.672)**	**<.001**	**.004**
Alcohol drinking	5.693 (−3.028, 14.415)	.201	**27.737 (3.508, 51.967)**	**.025**	.093
DBP (per 1 mmHg)	**0.405 (0.111, 0.698)**	**.007**	**0.387 (0.143, 0.632)**	**.002**	.924
Hypertension	**13.052 (3.038, 23.066)**	**.011**	2.294 (−6.073, 10.662)	.591	.105
Classification of hypertension^a^					.341
Grade 1	**11.025 (1.439, 20.612)**	**.024**	3.256 (−3.713, 10.226)	.360	
Grade 2	**8.122 (0.035, 16.209)**	**.049**	2.575 (−3.423, 8.574)	.400	
Grade 3	**19.021 (5.045, 32.996)**	**.008**	**22.522 (9.950, 35.094)**	**<.001**	
Antihypertensive medication	**−8.012 (−15.642, −0.381)**	**.040**	−2.122 (−8.448, 4.204)	.511	.243

*Note*: The linear regression model was adjusted for age, sex, baseline mean bilateral cIMT, smoking, BMI, SBP, lipids (TC and TG), comorbidities (hypertension, diabetes, dyslipidemia, and CVD) and medicine use (antihypertensive medication and lipid‐lowering medication). Bold values represent statistic significant *p*‐value < .05.

Abbreviations: CI, confidence interval; cIMT, carotid intima‐media thickness; DBP, diastolic blood pressure.

^a^ The association of classification of hypertension with annual cIMT change rate was analyzed with all the variables mentioned above adjusted expect for hypertension and SBP, using normotension as the reference.

For new plaque presence (Table 5), age and hypertension were significant risk factors in both sexes. But smoking (OR = 1.637, 95% CI: 1.225, 2.188), TG ≥ 1.7 mmol/L (OR = 1.342 95% CI: 1.004, 1.793) and dyslipidemia (OR = 1.541 95% CI: 1.134, 2.094) was significantly associated with new plaque presence only in men, TC ≥ 5.2 mmol/L (OR = 1.881, 95% CI: 1.489, 2.375) and diabetes (OR = 1.423, 95% CI: 1.103, 1.836) were only significant in women. other included variables had no significant associations with new plaque presence in both sexes. TC ≥ 5.2 mmol/L significantly promoted new plaque formation in women compared to men (*p* for interaction = 0.042).

**Table 5:** Association between risk factors and new carotid plaque presence by sex

Variables	New carotid plaque presence				
	Male		Female		
	OR (95% CI)	*p*‐value	OR (95% CI)	*p*‐value	*p* for interaction
Age (per 1 year)	**1.058 (1.041, 1.076)**	**<.001**	**1.074 (1.059, 1.089)**	**<.001**	.152
Smoking	**1.697 (1.263, 2.281)**	**<.001**	0.835 (0.308, 2.260)	.722	.165
TC ≥ 5.2 mmol/L	1.150 (0.784, 1.686)	.475	**1.874 (1.400, 2.507)**	**<.001**	**.042**
TG ≥ 1.7 mmol/L	**1.351 (1.008, 1.811)**	**.044**	1.098 (0.886, 1.361)	.392	.260
Hypertension	**1.546 (1.110, 2.154)**	**.010**	**1.386 (1.081, 1.775)**	**.010**	.565
Classification of hypertension^a^					.568
Grade 1	1.282 (0.828, 1.986)	.265	1.243 (0.908, 1.702)	.175	
Grade 2	**1.523 (1.059, 2.189)**	**.023**	1.236 (0.932, 1.638)	.141	
Grade 3	1.226 (0.649, 2.317)	.529	**2.170 (1.230, 3.828)**	**.007**	
Diabetes	1.055 (0.735, 1.516)	.771	**1.395 (1.075, 1.810)**	**.012**	.219
Dyslipidemia	**1.625 (1.036, 2.549)**	**.035**	0.992 (0.751, 1.310)	.953	.066

*Note*: The logistic regression model was adjusted for age, sex, baseline mean bilateral cIMT, smoking, alcohol drinking, overweight, TC ≥ 5.2 mmol/L, TG ≥ 1.7 mmol/L, comorbidities (hypertension, diabetes, dyslipidemia, and CVD) and medicine use (antihypertensive medication and lipid‐lowering medication). Bold values represent statistic significant *p*‐value < .05.

Abbreviations: CI, confidence interval; OR, odd ratio; TC, total cholesterol; TG, triglyceride.

^a^ The association of hypertension classification with new carotid plaque presence was analyzed separately with all the variables mentioned above adjusted expect for hypertension, using normotension as the reference.

## 4 DISCUSSION

In this study, we have identified risk factors for the progression of cIMT and carotid plaque and found different risk factors between men and women. Age, smoking, hypertension and diabetes were common risk factors for both cIMT progression and new plaque formation in all participants. For women, smoking and alcohol drinking were significantly related to cIMT progression, while lipids metabolism, hypertension and diabetes were related to plaque presence. For men, hypertension was significant risk factor for cIMT progression, while lipids metabolism, hypertension and smoking affected the formation of plaques.

The previous studies which explored the sex‐specific risk factors for carotid atherosclerosis usually focused on a specific category of indicators,^20^, ^21^ or did not concern the changes of cIMT and plaques.^22^, ^23^ Several follow‐up studies also compared a variety of sex‐specific risk factors with carotid atherosclerosis progression, but they used relatively smaller sample size.^24^, ^25^ In this study, we conducted a large‐scale screening for high‐risk population of CVD across communities and collected abundant information to investigate the risk factors. It was recognized that cIMT may reflect a wider range of risk factors than plaques, while plaques may reflect mainly atherosclerotic risk factors.^26^ We distinguished the risk factors for cIMT progression and plaque formation separately. The annual cIMT change rate of all participants signified a stable situation. We enrolled participants at high‐risk CVD and took interventions including suggesting quitting smoking or alcohol, taking more exercise and following healthy diets. We also offered guidance for medication. The inverse association of baseline cIMT with cIMT progression was consistent with previous studies.^24^, ^27^ Individuals with elevated cIMT at baseline had higher cardiovascular risk, thus may receive stricter intervention, which might explain why they had a regression of cIMT. At baseline, women had better carotid health conditions compared to men. Correspondingly, men presented more risk factors than women. Studies have confirmed that estrogen could optimize metabolism, macrophage or smooth muscle cell function, which help women to resist atherosclerosis.^28^ Sex‐specific genetic effects were also considered.^29^ Nevertheless, postmenopausal hormone replacement therapy was not proved to ultimately reduce the risk of atherosclerosis, which indicates biological mechanism of female hormones still needs to be further studied.^30^ Iemolo et al. found that sex hormones may influence the remodeling of atherosclerosis, causing more stenosis but less plaque presence in women than in men.^31^ Though women had more ideal health status at baseline, the cIMT change rate and newly diagnosed plaque presence rate were not significantly distinguished between sexes. Considering most of the females in our study were postmenopausal (71.00%), the protective effect of estrogen may have declined.

Smoking is universally known as a strong factor for ASCVD. This study has revealed the significant association of smoking with both cIMT progression and plaque formation, the impact of smoking on cIMT progression is much greater in women. A prospective study based on UK Biobank participants also found that smoking was related to higher risk for myocardial infarction in women.^32^ The greater susceptibility to smoking‐related endothelial dysfunction in women might explain.^33^ The associations between female smoking and plaque formation showed greater heterogeneity, which may be due to the small sample size of women who were smoking at baseline and without plaques. The association between alcohol consumption and atherosclerosis is controversial. Consistent with our study, high alcohol consumption is associated with increased cIMT.^34^ But other studies noted that a relatively lower dose of alcohol consumption was protective for cardiovascular health.^35^, ^36^ We did not find significant correlations for obesity index, but another follow‐up study found baseline BMI associated with cIMT progression just in men.^25^ Further studies evidenced that central fat mass was atherogenic, but peripheral fat mass was antiatherogenic, especially in elderly women.^37^

The association of dyslipidemia with carotid atherosclerosis progression is stronger in men. For lipid profiles, TC was a better predictor for new plaque formation in women, while TG predicted better in men. This result may contradict previous findings. Iso et al. have considered the effect of estrogen which could reduce the variation of TG.^38^ But most of the enrolled females were post‐menopausal and aged older in our study. Presented by the baseline characteristics, the cholesterol levels of women were higher than that of men, but TG is lower. Decrease in estrogen after menopause will lead to the increase in cholesterol, which accelerates the progression of atherosclerosis.^39^ Consistent with previous studies, CVD is strongly associated with cIMT progression.^6^ Hypertension and diabetes have been recognized as the common risk factors for artery atherosclerosis, favorable blood pressure and glucose control could slow the progression of carotid atherosclerosis.^40^, ^41^ Our results showed greater impact of hypertension on men, and men with grade 1 and grade 2 hypertension might experience higher degree of carotid atherosclerosis progression compared to women with the same grade, while women with grade 3 hypertension had higher degree of carotid atherosclerosis progression than men. Though men were more likely to respond to antihypertensive treatment for cIMT reversion, the antihypertensive treatment rate was higher in female hypertensive patients, and previous studies have shown a greater reduction of plaques in women with treated hypertension.^42^ Diabetes has a greater impact on women for plaque formation. Another prospective study had similar discovery that elevated hemoglobin A1c was associated with developing carotid plaque in women but not in men.^43^ Observational studies also demonstrated that women with diabetes had higher risk of CVD than male patients.^32^, ^44^ In diabetic patients, women may have lower plaque volumes but enhanced systemic inflammation, which may cause the increased risk of developing plaques and worse plaque composition.^45^

The limitations of this study should be noticed. First, we have a relatively short observation period. Second, the physical and laboratory examination data was only collected completely at baseline, we could not assess the changes of these data during the follow‐up. It is unclear how these changes of risk factors may affect the results. Third, the islet beta cell function and relevant autoimmune markers were not tested, so we could not further classify diabetes as type 1 and type 2. Moreover, for the analysis of new plaque formation, because the specific number of plaques is not collected, we treated plaque presence as a binary variable, which reduced accuracy. Due to the technical limitations, we could not assess more parameters like the total plaque area, which could be a more precise method for evaluation of carotid plaques.

## 5 CONCLUSIONS

In this study, we have identified risk factors for cIMT progression and plaque formation, sex‐specific risk factors were also revealed. For all participants, age, smoking, hypertension and diabetes were risk factors for both cIMT and plaque progression. But the risk factors differed by sex. Sex‐specific prevention and management strategies for carotid atherosclerosis are needed.

## CONFLICT OF INTEREST

The authors declare no conflict of interest.

### 1 DATA AVAILABILITY STATEMENT

The data used in this study can be obtained from the corresponding author upon reasonable request.

## Supporting information

Figurementary Figure 1 The research flow chart.

Supporting information.
