# Supplementary figures and images for: Sex‐specific risk factors of carotid atherosclerosis progression in a high‐risk population of cardiovascular disease
Source: Clin Cardiol. 2022 Oct 13;46(1):22–31. doi: 10.1002/clc.23931 (PMC9849433; doi:10.1002/clc.23931)

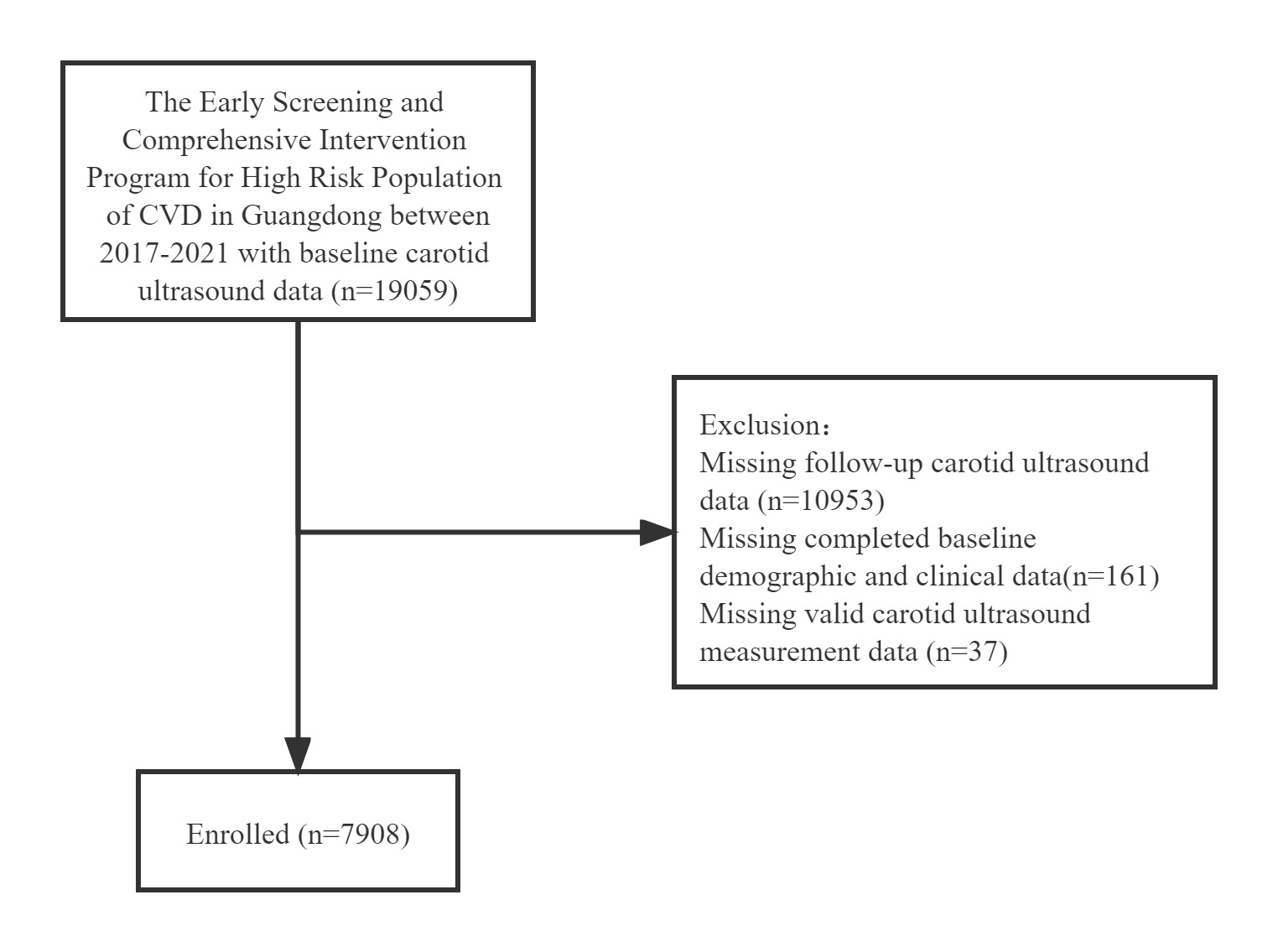

Supplement: Supplementary file 1 — Figurementary Figure 1 The research flow chart. [file CLC-46-22-s002.jpg]
