# Supplementary material for: Sex‐specific risk factors of carotid atherosclerosis progression in a high‐risk population of cardiovascular disease
Source: Clin Cardiol. 2022 Oct 13;46(1):22–31. doi: 10.1002/clc.23931 (PMC9849433; doi:10.1002/clc.23931)
Supplement: Supplementary file 2 — Supporting information. [file CLC-46-22-s001.docx]

**Supplementary Table 1. Baseline characteristics of the participants (classified by having plaque at baseline or not)**

|  | **Total** | **No plaque at**  **baseline** | **Have plaque at**  **baseline** | **P-value** |
| --- | --- | --- | --- | --- |
| Number | 7908 (100) | 4085 (51.66) | 3823 (48.34) |  |
| Demographics and Anthropometrics |  |  |  |  |
| Age, years | 57.75 ± 9.45 | 54.02 ± 9.13 | 61.73 ± 8.05 | <0.001 |
| Gender-female | 4864 (61.51) | 2750 (67.32) | 2114 (55.30) | <0.001 |
| Smoking, n (%) | 1405 (17.77) | 545 (13.34) | 860 (22.50) | <0.001 |
| Alcohol drinking, n (%) | 470 (5.94) | 201 (4.92) | 269 (7.04) | <0.001 |
| BMI,kg/m2 | 24.84 ± 3.41 | 24.75 ± 3.47 | 24.93 ± 3.35 | 0.003 |
| Waist circumference,cm | 86.19 ± 9.51 | 85.18 ± 9.64 | 87.28 ± 9.26 | <0.001 |
| Overweight, n(%) | 4617 (58.38) | 2310 (56.55) | 2307 (60.35) | <0.001 |
| Obesity, n(%) | 1310 (16.57) | 689 (16.87) | 621 (16.24) | 0.457 |
| Hemodynamics |  |  |  |  |
| Systolic blood pressure, mmHg | 142.32 ± 22.75 | 138.10 ± 22.76 | 146.83 ± 21.86 | <0.001 |
| Diastolic blood pressure, mmHg | 83.25 ± 13.19 | 82.99 ± 13.40 | 83.53 ± 12.94 | 0.016 |
| Heart rate, bpm | 78.64 ± 11.06 | 78.79 ± 10.84 | 78.48 ± 11.28 | 0.458 |
| Lipids and glucose, mmol/L |  |  |  |  |
| TC | 5.51 ± 1.49 | 5.46 ± 1.48 | 5.57 ± 1.50 | 0.001 |
| LDL-C | 3.28 ± 1.31 | 3.23 ± 1.31 | 3.33 ± 1.32 | <0.001 |
| HDL-C | 1.48 ± 0.48 | 1.49 ± 0.49 | 1.47 ± 0.46 | 0.043 |
| TG | 1.88 ± 1.09 | 1.86 ± 1.12 | 1.91 ± 1.07 | <0.001 |
| Non-HDL-C | 4.03 ± 1.37 | 3.96 ± 1.36 | 4.10 ± 1.38 | <0.001 |
| Glucose | 6.13 ± 1.80 | 5.96 ± 1.60 | 6.30 ± 1.98 | <0.001 |
| Comorbidities, n (%) |  |  |  |  |
| Hypertension | 4883 (61.75) | 2152 (52.68) | 2731 (71.44) | <0.001 |
| Classification of hypertension |  |  |  | <0.001 |
| Grade 1 | 1207 (15.26) | 535 (13.10) | 672 (17.58) |  |
| Grade 2 | 2758 (34.88) | 1232 (30.16) | 1526 (39.92) |  |
| Grade 3 | 377 (4.77) | 166 (4.06) | 211 (5.52) |  |
| Diabetes | 1712 (21.65) | 703 (17.21) | 1009 (26.39) | <0.001 |
| Dyslipidemia | 3391 (42.88) | 1671 (40.91) | 1720 (44.99) | <0.001 |
| Coronary heart disease | 190 (2.40) | 55 (1.35) | 135 (3.53) | <0.001 |
| History of myocardial infarction | 80 (1.01) | 28 (0.69) | 52 (1.36) | 0.003 |
| History of stroke | 137 (1.73) | 52 (1.27) | 85 (2.22) | 0.001 |
| CVD | 322 (4.07) | 106 (2.59) | 216 (5.65) | <0.001 |
| Treatment, n (%) |  |  |  |  |
| Antihypertensive medication | 2510 (31.74) | 966 (23.65) | 1544 (40.39) | <0.001 |
| Hypoglycemic medication | 688 (8.70) | 235 (5.75) | 453 (11.85) | <0.001 |
| Lipid-lowering medication | 408 (5.16) | 143 (3.50) | 265 (6.93) | <0.001 |
| Baseline cIMT and plaques |  |  |  |  |
| Baseline mean bilateral cIMT, mm | 0.79 ± 0.15 | 0.72 ± 0.13 | 0.82 ± 0.15 | <0.001 |
| Baseline increased cIMT, n(%) | 2125 (26.87) | 626 (15.32) | 1499 (39.21) | <0.001 |
| Baseline plaque thickness on the left side, mm | 2.16 ± 1.01 | NA | 2.16 ± 1.01 |  |
| Baseline plaque thickness on the right side, mm | 2.17 ± 0.93 | NA | 2.17 ± 0.93 |  |

Abbreviations: n, number; NA, not available; BMI, body mass index; TC, total cholesterol; LDL-C, low-density lipoprotein cholesterol; HDL-C, high-density lipoprotein cholesterol; TG, triglyceride; non-HDL-C, non-high-density lipoprotein cholesterol; CVD, cardiovascular disease; cIMT, carotid intima-media thickness.

Values are presented as mean ± standardized differences or n (%).
